# The Oxford Utilitarianism Scale: Psychometric properties of a French adaptation (OUS-Fr)

**DOI:** 10.3758/s13428-023-02250-x

**Published:** 2023-10-04

**Authors:** Robin Carron, Nathalie Blanc, Royce Anders, Emmanuelle Brigaud

**Affiliations:** Department of Psychology, EPSYLON Laboratory UR4556, University Paul Valéry, Montpellier 3, F34000 Montpellier, France

**Keywords:** Oxford Utilitarianism Scale, Moral dilemmas, Convergent validity, Confirmatory factor analysis

## Abstract

It is well established that one’s sense of morality may be readily influenced by one’s culture, education, and life situation. Very few psychometric tools are currently available to measure facets of human morality in different cultures. Therefore, the purpose of the present study was to develop a French adaptation of the Oxford Utilitarianism Scale (OUS-Fr) and formally evaluate its validity. The OUS-Fr was developed through a process of back-translation and administered to a sample of 552 participants. Results from exploratory factor analyses revealed a bidimensional structure with satisfactory loadings that was then also supported in the confirmatory factor analysis check. The OUS-Fr scale demonstrated good psychometric properties, with acceptable internal consistency and coherent results in the convergent validity analyses. These findings contribute to morality measurement literature by providing evidence for the reliability and validity of the French adaptation of the OUS. The OUS-Fr can be viewed as a valuable tool for researchers and practitioners for assessing utilitarian tendencies within the French-speaking population, which could pave the way for cross-cultural understandings that are important for fully understanding the intricacies of human morality.

## Introduction

Utilitarianism, a hallmark theory in human ethics, may be fundamentally characterized by the premise that the highest virtue equates to the greatest good for the greatest number of people. Its origins can be traced back to Jeremy Bentham and John Stuart Mill in the eighteenth and nineteenth centuries, respectively. This “*consequentialist*” approach to ethics is concerned primarily with the outcomes, or consequences of actions, placing an emphasis on achieving the maximal net happiness or pleasure. In other words, utilitarianism holds that an action is morally right, or optimal, if it results in more total happiness than any other alternative action. Bentham's version of utilitarianism (1781), often referred to as classical utilitarianism, operates on a quantitative measure of happiness, where the right action is determined by the one that generates the most amount of pleasure and the least amount of pain. On the other hand, Mill (1863) refined the theory to distinguish between higher and lower forms of pleasure, advocating for quality over quantity.

Since Jeremy Bentham, utilitarianism continues to be valued yet notably debated in philosophy works. However, despite different points of view, this ethical theory remains an exemplary doctrine in the fields of moral philosophy and moral psychology. It has stimulated reflections on how to maximize collective well-being through actions and has inspired debate on fundamental ethical and moral questions.

### Beyond moral dilemmas

The majority of research exploring utilitarianism has relied primarily on the paradigm of sacrificial dilemmas (Rest, 1994), such as the trolley dilemma (Foot, 1967). This paradigm places individuals in hypothetical situations where they are faced with the difficult decision of sacrificing one person's life in order to save the lives of a greater number. These moral dilemmas highlight the conflict between utilitarian (Mill, 1863) and deontological judgments (Kant, 1785). Choosing the sacrificial option involves a utilitarian approach that prioritizes the greatest good for the greatest number, while rejecting this possibility reflects a deontological judgment, which is based on respecting established moral rules (not taking a person’s life, regardless of any “net” gain).

However, in the last decade or so, recent research has been highly critical of this tool. The sacrificial dilemma approach has been criticized for its insufficiency to reliably assess the ethical concepts that some researchers claim it evaluates. Kahane (2015) states that a sacrificial dilemma is unable to assess utilitarianism and deontology, because in order to solve the problem, subjects use neither utilitarian nor deontological morality, but rather a “common sense” morality emphasizing justice and fairness (Baumard & Sheskin, 2015). Furthermore, this particular approach to the study of utilitarianism raises concerns because the utilitarian judgments made in moral sacrificial dilemmas do not accurately reflect an impartial concern for the greater good. More specifically, the sacrifice of an individual in a trolley-type dilemma is not necessarily motivated by a genuine concern for the greater common good. Indeed, Kahane (2015) and Kahane et al. (2018) have demonstrated that individuals with antisocial personality disorder have a tendency to make utilitarian decisions in sacrificial dilemmas, not driven by a genuine concern for maximizing the number of lives saved, but rather influenced by their personal inclination towards sacrifice. Given these criticisms, moral dilemmas, as they are used in scientific literature so far, fall short of allowing for a proper assessment of the utilitarian dimension of human morality. In particular, Kahane et al. (2018) explain that the dilemma paradigm seems unable to capture the complexity of utilitarianism. Therefore, they introduced a novel conceptual framework for the study of utilitarianism: the two-dimensional (2D) model.

### The two dimensional (2D) model

This model distinguishes two key dimensions of utilitarianism: the negative dimension, instrumental harm, and the positive dimension, impartial beneficence (Kahane et al., 2018). Instrumental harm refers to causing harm in order to achieve a greater good. According to this perspective, while it is crucial to prioritize honesty, honor commitments, and protect the innocent, it is important to acknowledge that there are situations where deviating from these established moral principles becomes necessary to maximize overall well-being and accomplish the greatest benefit for the majority. It is observed that research exploring the paradigm of dilemmas primarily focuses on the instrumental harm dimension of utilitarianism (Bartels & Pizarro, 2011; Kahane, 2015). Indeed, the majority of sacrificial dilemmas presented require sacrificing an individual to save a greater number. This is exemplified by the well-known bridge dilemma (Thomson, 1984), in which one is asked to push an individual onto the train tracks to save five people. On the other hand, impartial beneficence refers to promoting the greater good, even at the cost of self-sacrifice, knowing that the well-being of each individual is important (e.g., donating a kidney to a person suffering from renal failure). Therefore, according to utilitarian principles, we should treat the well-being of each individual equally, regardless of the status, relationship, or proximity, even if it entails self-sacrifice. Taking an absolutely utilitarian moral perspective, we should approach the interests of all individuals uniformly regardless of personal, emotional, spatial, or temporal distance. According to this dimension, it is paramount not to favor our own interests or those of our family, friends, fellow citizens, or even our fellow beings.

The logic underlying this model aims to rethink our understanding of utilitarianism. Given that utilitarian reasoning is multidimensional in nature, it would be incorrect to categorize a judgment or an individual as strictly utilitarian or non-utilitarian. On the contrary, according to this model, judgments and individuals should be described through the various facets of utilitarianism. As Everett and Kahane (2020) state, pro-sacrificial judgments may reflect endorsement of instrumental harm but not greater impartiality, whereas the reverse may be true of judgments endorsing sacrifices in aid of distant strangers.

### A new tool: the OUS

Using this conceptual framework, Kahane et al. (2018) developed and validated the Oxford Utilitarianism Scale (OUS), designed to address significant limitations of the moral dilemmas paradigm. This scale aims to analyze individual variations regarding proto-utilitarian tendencies by probing the positive and negative dimensions of utilitarianism. The authors seek to build a scale that will assess the degree to which individuals' moral intuitions, as manifested through judgments, align or diverge from utilitarianism.

The OUS is presented as a self-evaluation scale composed of nine items, with five items related to impartial beneficence (OUS-IB) and four items related to instrumental harm (OUS-IH). The measures obtained through these subscales have successfully delineated the characteristics of the two dimensions. Individuals who score highly on both the OUS-IB and OUS-IH scales are more inclined to favor utilitarian solutions in sacrificial moral dilemmas (Kahane et al., 2018; Körner et al., 2020). According to utilitarian logic, the preference for utilitarianism should result from a focus on consequences. However, Körner et al. (2020) demonstrated that this preference for utilitarianism is driven more by a reduced sensitivity to norms rather than an enhanced focus on consequences. This interpretation aligns with the findings on psychopathy. A high score on the OUS-IB scale correlates with a low level of psychopathy, while a high score on the OUS-IH scale is indicative of a high level of psychopathy (Kahane et al., 2018). As demonstrated by multiple studies (Paruzel-Czachura & Farny, 2023; Gawronski et al., 2017; Luke & Gawronski, 2021), individuals with high levels of psychopathy exhibit an impaired understanding of societal moral norms and show insensitivity to feelings of guilt. This understanding of psychopathy is further complemented when considering the relationship between these scales and empathic concern. A high score on the OUS-IB scale correlates with a high level of empathic concern, and is associated with heightened generosity and deeper identification with humanity at large. In contrast, a high score on the OUS-IH scale is inversely correlated to empathic concern (Kahane et al., 2018; Paruzel-Czachura & Farny, 2023). Overall, these results on the OUS subscales provide a nuanced insight into the complex interplay between psychopathy and empathic concern in individuals.

If these results seem to demonstrate consistent construct validity of the OUS, other findings may raise questions regarding the complementarity of these two dimensions. While a number of studies have shown a positive correlation between the OUS-IB and OUS-IH scores (e.g., Kahane et al., 2018, study 2; Paruzel-Czachura & Kocur, 2023; Park et al., 2023; Navajas et al., 2021), others did not find a significant correlation between them (Filiz & Hasan, 2021; Kahane et al., 2018, study 1). Such results could mean that the two dimensions are related but distinct constructs, and play different roles in the influence of moral judgment. Also, correlations between the OUS-IB and OUS-IH scores may be influenced by various intrinsic and contextual factors. Variations in levels of emphatic concern and psychopathy within studied samples can affect these correlations, especially if a particular population exhibits a higher rate of psychopathy. Moreover, as highlighted by Körner et al. (2020), inclinations toward utilitarianism might be more driven by a reduced sensitivity to norms than an actual utilitarian preference. This sensitivity could, in turn, vary across cultures or groups, thereby altering the way individuals respond to the OUS.

### More than a scale, a new framework

Never has moral psychology been in the spotlight more than today. Between 1940 and 2017, Ellemers et al. (2019) identified a total of 1278 articles addressing topics of morality, with the publication rate notably increasing over the decades. For example, since 2014, over 100 articles have been published per year on moral psychology versus only 10 publications in 1981. Although research production on moral psychology is thriving today, surprisingly, many studies continue to rely on very dated methods such as *the classic trolley dilemma*, devised over 50 years ago (Foot, 1967). While this conservative reluctance to embrace newer methods can be explained, in part, by the complexity associated with studying morality appropriately, it can also be argued in part due to the lack of a common framework that both proves to be empirically suitable and manages to be commonly accepted by the community.

The recent replication crisis (Open Science Collaboration, 2015), in combination with research that has criticized traditional tools in moral psychology such as the previously mentioned trolley dilemma (see Kahane, 2015; Bauman et al., 2014; Dahl & Oftedal, 2019; Carron et al., 2022), has engendered a movement to revisit the scientific integrity of moral psychology and current methodologies (Ellemers et al., 2019). Especially in light of the replication crisis in psychology that is increasingly taken seriously and in which studies are scrutinized for their empirical soundness, it is of paramount importance for moral psychology to cohere and develop the best scientific practices possible.

Because some major critiques of sacrificial dilemmas emerged, it should be noted that before the introduction of the OUS, researchers had already worked on developing more sophisticated tools. These tools, in the form of self-report measures, utilize experimental and personological psychology methodologies. A diverse array of tools exists for assessing human morality, and the following list provides a non-exhaustive selection of these instruments: *the Moral Foundation Questionnaire* (Graham et al., 2011), *the Moral Foundations Vignettes* (Clifford et al., 2015), *the Ethical Values Assessment* (Padilla-Walker & Jensen, 2016), *the Moralization of Everyday Life Scale* (Lovett et al., 2012), *the Self-Importance of Moral Identity Scale* (Aquino & Reed, 2002), and more recently *the OUS* (Kahane et al., 2018). However, the introduction of the OUS represents more than a new tool in the researcher's arsenal; it signifies a paradigm shift in the study of moral psychology. The 2D model underlying the OUS reframes our understanding of utilitarianism, emphasizing its multidimensional nature and prompting us to reconsider how we conceptualize and assess moral judgment. By recognizing and evaluating the dual dimensions of instrumental harm and impartial beneficence, the OUS promotes a more nuanced exploration of moral psychology. This novel framework encourages a departure from a binary utilitarian/non-utilitarian classification towards a spectrum of moral inclinations. The OUS's effectiveness and pioneering approach are reflected in its wide acceptance and rapid integration into diverse research programs. A literature review (conducted using Web of Science [WoS]) indicated that the OUS has already been cited in 137 publications to date, of which 10% implemented the scale. The scale has already been translated and validated in one other language, that is, Turkish (Filiz & Hasan, 2021). Additionally, there is a Polish version of the OUS (Paruzel-Czachura et al., 2023) that has yet to be formally validated with the typical analyses expected for language adaptations. Specifically, the OUS was translated into Polish for experimental purposes, in order to investigate whether alcohol increased the utilitarianist facet in moral judgments.

Given that French is the fifth most spoken language with more than 321 million speakers predominantly in France, Canada, Africa, and Switzerland, and that the OUS is beginning to have a consequential impact in moral psychology research, the validation of this new scale in French is important both towards endorsing a common framework as mentioned previously, and in appropriately addressing the replication crisis in psychology.

## Method

### Participants

Our study involved a wide range of participants that were not solely of a student population. Participants were recruited online on social media platforms as well as through local announcements to the students of the university. The demographic variables collected from the participants consisted of their age, gender, and educational background. The inclusion criteria consisted of the following: 18 years of age or older, fluent in French, and able to provide informed consent. Participants completed the study online from their own devices, were not paid for their participation, and their participation was entirely voluntary. This study was conducted in accordance with the ethical guidelines set forth by the ethics committee of Paul Valéry University, Montpellier III, under Institutional Review Board (IRB) protocol number IRB00013686- 2023-07-CER. All participants provided informed consent prior to their participation in the study, and all procedures were approved by the ethics committee.

## Translation

The original scale was developed in English. The translation process followed the guidelines proposed by Beaton et al. (2000) for cross-cultural adaptation of self-report measures. Additionally, more recent work that revisited these guidelines was considered (Cha et al., 2007; Fenn et al., 2020; Sousa & Rojjanasrirat, 2011; Tsang et al., 2017). Notably, these studies provided valuable insights into the best practices involved in translating scales across languages and cultures. In accordance with these guidelines, the translation process involved the following principal steps:

- *Forward translation:* Three independent bilingual translators, whose native language was French and who were fluent in English (C1 level; Council of Europe, n.d.), translated the scale from English to French. They were instructed to produce a version that was conceptually equivalent to the original and culturally appropriate for French-speaking individuals.
- *Synthesis:* The three French versions were compared and reconciled by a committee of experts that included the translators and the research team. The committee aimed to resolve any discrepancies between the translations and to produce a consensual version that reflected the intended meaning of the original scale.
- *Backward translation:* A fourth bilingual translator, whose native language was French and who was fluent in English, translated the French version back into English. This step aimed to ensure that the French version was still conceptually equivalent to the original scale and that no important information was lost during the translation process.

Overall, the translation process followed a rigorous and systematic approach that aimed to ensure the equivalence and cultural appropriateness of the French version of the scale. We believe it is necessary to specify that this version is designed for French people and would require another validation for other French-speaking populations.

## Materials

As part of validating the OUS in French and relating it to other relevant scales, we exclusively utilized the scales that were also present in the original study by Kahane et al. (2018), and had also previously been validated in French (see Table 1).

**Table 1:** Scales used in the study

Measure	Scale rating	M	SD	Min	Max
Overall Oxford Utilitarianism	1–7	3.70	0.89	9	63
Impartial beneficence	1–7	4.10	1.17	4	28
Instrumental harm	1–7	3.19	1.26	5	35
Psychopathy	1–4	1.98	0.39	16	64
Empathic concern	1–5	4.02	0.61	7	35
Need for cognition	1–4	2.75	0.59	11	44

## The OUS

The OUS is a self-report tool designed to measure an individual's utilitarian ethical beliefs and values (Kahane et al., 2018). It consists of nine items, rated on a seven-point Likert scale ranging from 1 = *strongly disagree* to 7 = *strongly agree*. The OUS comprises two subscales respectively measuring the “instrumental harm” dimension related to utilitarianism (OUS-IH), which includes four items, and the “impartial beneficence” dimension (OUS-IB), which includes five items. In this scale, it is possible to find items such as “It is morally right to harm an innocent person if that harm is a necessary means to help many other innocent people?” for the OUS-IH, and “If the only way to save another person’s life during an emergency is to sacrifice one’s own leg, then one is morally bound to make this sacrifice,” for the OUS-IB. In their study, Kahane et al. (2018) reported an excellent model fit—specifically, for instrumental harm: *χ*^2^(2) = 3.56, *p* = .16, Comparative Fit Index (CFI)= .99, Root Mean Square Error of Approximation (RMSEA) = .053 [.00, .14], Standardized Root Mean Square Residual (SRMR) = .02, Akaike Information Criterion (AIC)= 4027.46, Bayesian Information Criterion (BIC) = 4056.59, and for impartial beneficence: *χ*^2^(5) = 3.75, *p* = .59, CFI = 1.00, RMSEA = .00 [.00, .07], SRMR = .02, AIC = 5281.13, BIC = 5317.54. These goodness-of-fit indicators from the Confirmatory Factor Analysis (CFA) results support the validity of the two dimensions of the OUS, “instrumental harm” and “impartial beneficence,” according to the expected norms (e.g., Hu & Bentler, 1999; Hooper et al., 2008). That is, the idea that the items in the OUS that are respectively intended to measure these two constructs is corroborated by the factor analysis.

## Levenson’s primary psychopathy subscale

This self-report tool was developed by Levenson et al. (1995) and later validated in French by Savard et al. (2014). In our study, we utilized the primary psychopathy subscale of the Levenson Self-Report Psychopathy Scale (LSRP), which is a widely used measure of psychopathy that also contains a secondary psychopathy subscale. The primary psychopathy subscale includes a 16-item subscale that assesses various aspects such as manipulativeness, impulsivity, and narcissism. In this scale, it is possible to find items such as “Success is based on survival of the fittest; I am not concerned about losers.” Participants rate their agreement with each item on a four-point scale, ranging from 1 = *strongly disagree* to 4 = *strongly agree*. Higher scores on the primary psychopathy subscale indicate greater levels of primary psychopathy. Items 10, 12, 14, 15, and 16 are reversed before rating.

## Empathic Concern subscale of the Interpersonal Reactivity Index

The Empathic Concern subscale of the Interpersonal Reactivity Index (IRI) was used to measure participants' empathy levels. The IRI was developed by Davis (1980) and translated into French by Gilet et al. (2013). It consists of four subscales: Perspective Taking, Fantasy, Empathic Concern, and Personal Distress. The Empathic Concern subscale, which is composed of seven items, assesses an individual's tendency to experience feelings of compassion and concern for others in need. Exemplary items are “I often have tender, concerned feelings for people less fortunate than me” and “When I see someone being taken advantage of, I feel kind of protective towards them.” Participants rated their agreement with each item on a five-point scale, ranging from 1 = *Does not describe me well* to 5 = *Describes me very well*. Higher scores on the Empathic Concern subscale indicate greater levels of empathy towards others. Items 2, 4, and 5 are reversed before aggregating the item response scores.

## Need for cognition

In our study, we assessed participants’ Need For Cognition (NFC) using the self-report tool developed by Cacioppo et al. (1996). The original version of the scale consists of 18 items, but we used the 11-item French version of the scale, which was reduced by Salama-Younes et al. (2014) and validated in French. The NFC scale measures the extent to which individuals are inclined to engage in and enjoy effortful cognitive activities, such as thinking critically or solving problems. Participants rate their agreement with each item on a four-point scale, ranging from 1 = *completely false* to 4 = *completely true*. For example, one item states, “I really enjoy a task that involves coming up with new solutions.” Higher scores on the NFC scale indicate a greater inclination to engage in effortful thinking. Items 3, 4, 8, and 11 are reversed before rating.

## Sociodemographic information

To gather information on gender, participants were asked to select “male” or “female” as their gender identity. Age was collected as a continuous variable, with participants providing their age in years. Education level was collected by asking participants to select their highest level of education completed from the following options: middle school, high school, junior college, technical school, bachelor's degree, master’s degree, and doctoral degree. After collecting the data on education levels using the options mentioned above, we categorized the participants into two groups: those with a college education (technical school, junior college, bachelor's, master’s, and doctoral degrees) and those without, which formed two approximately equal-sized groups.

## Procedure

The procedure for this study was conducted online using the Qualtrics platform (https://www.qualtrics.com). Participants were informed that the aim of the research was to validate the French translation of the psychology scale. They were required to complete the experiment in a single, 15-minute session on an Internet-connected device. If they failed to do so, they were removed from the study. Each questionnaire was presented in its entirety on the screen, and they were instructed to click the “next” button to proceed to the next one. It was emphasized that they could not go back to previous questionnaires once they had answered them. Participants were also informed that their data would be processed anonymously and they had the right to withdraw from the study at any time. Before beginning the study, electronic consent was obtained from each individual. In order to ensure that participants understood the instructions, each questionnaire was translated into French.

To minimize any potential carryover effects and maintain the primary variable of interest, the OUS was consistently presented as the first scale. For this scale, participants were asked to indicate the extent to which they agreed or disagreed with each statement on a seven-point scale ranging from 1 = *strongly disagree* to 7 = *strongly agree*.

The remaining three scales were presented in random order to prevent order effects.

Finally, participants provided sociodemographic information, including gender, age, and educational level, to allow for later analysis of potential demographic influences. Overall, using Qualtrics allowed us to standardize the administration of the study, ensuring accurate data collection and analysis.

All statistical analyses were performed using JASP and RStudio. In our study, all of the response scales were ordinal variables, meaning that the data collected were of ordered categories. The assessment of construct validity involved conducting exploratory and confirmatory factor analyses. For the Exploratory Factor Analysis (EFA), we utilized the minimum residual method with varimax rotation and set the eigenvalue criterion to greater than 1 (Costello & Osborne, 2005). To validate the factor solutions, we utilized both scree plot inspections and parallel analyses. These methods were used to help us determine the optimal number of factors to extract and ensure that the factors were meaningful and not simply the result of chance. The CFA were conducted using the *lavaan* package in RStudio (Rosseel, 2012) via a maximum likelihood optimization method. Prior to testing, goodness-of-fit indices were evaluated following the CFA recommendations in the literature (Hu & Bentler, 1999; Hooper et al., 2008), which can be found in Table 3. The internal consistency of the French version of the OUS scale was assessed by calculating Cronbach's alpha and McDonald's omega for each subscale. Cronbach's alpha and McDonald's omega values greater than .70 are considered to indicate good internal consistency (Nunnally, 1978). Convergent validity was determined using Pearson’s correlations between the overall OUS, OUS-IB, OUS-IH, psychopathy, empathic concern, and need for cognition. We used Bonferroni-corrected *p*-values for multiple comparisons to control for the family-wise error rate in our statistical analysis. To explore the predictability of the subscales, multiple linear regression analyses using the stepwise method were performed to examine the association between the independent variables and the OUS-IB and OUS-IH.

## Results

### Population sample

The final sample consisted of 552 participants who satisfied the inclusion criteria, 457 women and 95 men with a mean age of 21.91 years (*SD* = 7.56). Of these participants, 346 had completed some form of higher education, while 206 had not.

## Exploratory factor analysis

Bartlett's test of sphericity (*χ*^2^= 956.39, *p* < .001) and the Kaiser–Meyer–Olkin (KMO) measure of sampling adequacy (KMO = .72) indicated that the correlation matrix was suitable for factor analysis. The scree plot indicated that a two-factor solution was appropriate, as the plot leveled off after the second factor. The first factor had an eigenvalue of 2.40 and accounted for 19.70% of the total variance, while the second factor had an eigenvalue of 2.08 and explained 17.30% of the variance. The remaining factors had eigenvalues less than 1. Thus, the two-factor model accounted for 37% of the total variance (Table 2). A table detailing the mean and standard deviation for each OUS item, along with their respective floor and ceiling effects, is available in the supplementary materials.

**Table 2:** OUS exploratory factor analysis and factor loadings

	OUS-IH	OUS-IB
IB-1	-	0.52
IB-2	-	0.71
IB-3	-	0.51
IB-4	-	0.40
IB-5	-	0.57
IH-1	0.59	-
IH-2	0.44	-
IH-3	0.77	-
IH-4	0.79	-

***Note*.** Applied rotation method is varimax

## Factor structure analysis

A CFA was conducted with the following data. Two models were tested, one with a single factor and the other with two factors (Table 3). Results showed that the two-factor model outperformed the one-factor model, thus supporting its selection as the preferred model (CFA: *χ*^2^(26) = 90.58, *p* < .001, CFI = .93, RMSEA = .07; 90% CI [.05, .08], SRMR = .05). All items showed strong loading values (λ > .50, *p* < .01).

**Table 3:** The confirmatory factor analysis results for two-factor solution of the OUS

	*χ*^2^	*df*	χ^2^/*df*	CFI	GFI	RMSEA	IFI	SRMR
**Two-factor model**	90.58	26	3.48	.93	.99	.07	.93	.05
**One-factor model**	570.89	27	21.14	.41	.96	.19	.42	.14
**Recommended** **value**				> .90	> .90	< .10	> .90	< .05

## Reliability: internal consistency

Regarding the internal consistency reliability, it can be stated that typically a Cronbach's alpha of .70 or higher is recommended (Table 4). These results rather gravitate around this value and suggest that the consensus is weaker in the OUS-IB than in the OUS-IH.

**Table 4:** Internal consistency for all scales

Scale	α	ω
Overall Oxford Utilitarianism Scale	.64	.60
Impartial beneficence	.67	.68
Instrumental harm	.73	.74
Psychopathy	.69	.68
Empathic concern	.70	.71
Need for cognition	.82	.82

## Convergent validity

We examined the correlations between the OUS and its subscales with other measures of related constructs, including psychopathy, empathic concern, and need for cognition (Table 5). The OUS total was positively correlated with impartial beneficence (*r* = .78, *p* < .01), instrumental harm (*r = *.69, *p* < .01), and psychopathy (*r* = .18, *p* < .001). Impartial beneficence correlated with empathic concern (*r* = .29, *p* < .01). Instrumental harm correlated with psychopathy (*r* = .28, *p* < .01), empathic concern (*r* = −.15, *p* < .01). Psychopathy correlated with empathic concern (*r* = −.27, *p* < .01), need for cognition (*r* = −.15, *p* < .01).

**Table 5:** Correlations between the OUS and other measures of utilitarianism

Measure	1	2	3	4	5
1. Overall Oxford Utilitarianism Scale	_				
2. Impartial beneficence	.78***	_			
3. Instrumental harm	.69***	.07	_		
4. Psychopathy	.18***	.01	.28***	_	
5. Empathic concern	**.11**	.29***	−.15**	−.27***	
6. Need for cognition	−.02	−.03	−.06	−.15**	.04

***Note*.** Asterisks indicate significance after Bonferroni correction * *p* < .05; ** p < .01; *** *p* < .001, with boldface indicating significance before a Bonferroni adjustment (*p* < .01).

## Discussion

### Main findings

The aim of the present study was to validate a French adaptation of the OUS (Kahane et al., 2018), provided in Table 6, following the suggested guidelines for adapting study instruments across different cultures (Beaton et al., 2000; Cha et al., 2007). Additionally, the study aimed to contribute to the current data available on the OUS and examine the potentially different outcomes that are possible in a French sample. This intention was inspired by the observation of significant disparities in convergent validity highlighted in the Turkish translation of the OUS (Filiz & Hasan, 2021). Overall, the results of our study which evaluated the internal consistency, construct validity, convergent validity, and predictive ability of the scale with a French population after translation, confirm the psychometric validity of the scale. Furthermore, we observed that the OUS total score and its two subscales demonstrated acceptable levels of internal consistency. Finally, the scale's convergent validity was consistent with the findings of Kahane et al. (2018).

**Table 6:** Item wordings and French translations

**Subscale 1. OUS-IB**	
*IB-1*	If the only way to save another person’s life during an emergency is to sacrifice one’s own leg, then one is morally required to make this sacrifice. / *En cas d'urgence, si le seul moyen de sauver la vie d'une personne est de sacrifier sa propre jambe, alors on est moralement tenu de faire ce sacrifice.*
*IB-2*	From a moral point of view, we should feel obliged to give one of our kidneys to a person with kidney failure since we do not need two kidneys to survive, but really only one to be healthy. / *D'un point de vue moral, nous devrions nous sentir obligés de donner un de nos reins à une personne souffrant d'insuffisance rénale, car nous n'avons pas besoin de deux reins pour survivre, mais bien d'un seul pour vivre en bonne santé.*
*IB-3*	From a moral perspective, people should care about the well-being of all human beings on the planet equally; they should not favor the well-being of people who are especially close to them either physically or emotionally. / *D'un point de vue moral, les gens devraient se soucier de la même manière du bien-être de tous les êtres humains ; ils ne devraient pas privilégier le bien-être des personnes qui leur sont particulièrement proches, que ce soit physiquement ou émotionnellement.*
*IB-4*	It is just as wrong to fail to help someone as it is to actively harm them yourself. / *Ne pas venir en aide à quelqu’un est aussi inacceptable que de lui faire du mal intentionnellement*.
IB-5	It is morally wrong to keep money that one doesn’t really need if one can donate it to causes that provide effective help to those who will benefit a great deal. / *Il est moralement condamnable de garder pour soi de l'argent dont on n'a pas réellement besoin, si l’on peut le donner pour soutenir des causes qui œuvrent effcacement auprès de ceux qui en ont vraiment besoin.*
**Subscale 1. OUS-IH**	
*IH-1*	It is morally right to harm an innocent person if harming them is a necessary means to helping several other innocent people. / *Il est moralement acceptable de faire du mal à une personne innocente si cela s’avère nécessaire pour aider plusieurs autres personnes innocentes.*
*IH-2*	If the only way to ensure the overall well-being and happiness of the people is through the use of political oppression for a short, limited period, then political oppression should be used. / *Si le seul moyen d'assurer le bien-être et le bonheur de la population est de recourir, pendant une courte période, à l'oppression politique, alors il faut le faire.*
*IH-3*	It is permissible to torture an innocent person if this would be necessary to provide information to prevent a bomb going off that would kill hundreds of people. / *Il est acceptable de torturer une personne innocente si cela est nécessaire pour obtenir en retour des informations empêchant l'explosion d'une bombe qui tuerait des centaines de personnes.*
*IH-4*	Sometimes it is morally necessary for innocent people to die as collateral damage—if more people are saved overall. / *Parfois, il est moralement nécessaire que des innocents meurent en tant que dommages collatéraux - si, au total, cela permet de sauver davantage de gens.*

First, the EFA results showed that a two-factor solution was appropriate, as supported by the scree plot analysis. The two factors (impartial beneficence and instrumental harm) accounted for a total of 37% of the total variance. To confirm the validity of the scale, we introduced two models: one with a single factor considering the scale as a global construct, and the other with two dimensions based on EFA and Kahane's original study. The calculated fit indices indicated that the two-dimensional model was a better fit (CFA: *χ*^2^(26) = 90.58, *p* < .001, CFI = .93, IFI = .93, RMSEA = .07; 90% CI [.05, .08], SRMR = .05).

Second, the internal consistency of the OUS total score and its two subscales was found to be acceptable (α = .64 for the total score, α = .67 for the OUS-IB and α = .73 for the OUS-IH). Although the internal consistency values for the OUS total score and the OUS-IB were found to be below the recommended threshold of .70 for Cronbach's alpha, it is not uncommon to encounter lower values of Cronbach's alpha during the process of validating a scale in another language, particularly when there are cultural and linguistic differences between the original and the translated versions. According to Schmitt (1996), it has been argued that a reliability coefficient as low as .50 should not seriously attenuate validity. Additionally, Miller (1995) suggests that the alpha coefficient tends to increase with the length of the instrument. In light of these considerations, these coefficient values may be considered compatible despite that more optimal magnitudes would have been preferred.

Thirdly, unlike the Turkish validation (Filiz & Hasan, 2021), the convergent analysis of the French scale is very close to the original. Our results reveal only a few discrepancies from Kahane et al.’s article (2018), as we found a positive correlation between the OUS and empathic concern, and no correlation between the OUS-IB and OUS-IH. As we suggested in the introduction, correlations between the OUS-IB and OUS-IH scores may be influenced by various contextual factors, especially cultural factors. Nevertheless, our convergent analysis aligns with Kahane's initial findings. Consistent with their conclusions, we observed that the OUS-IB was positively associated with empathic concern, while the OUS-IH was positively associated with psychopathy and negatively associated with empathic concern. This suggests that individuals with higher levels of psychopathy are more likely to harm others and less likely to maximize others' well-being. These findings are consistent with the results of Paruzel-Czachura and Farny (2023), which showed that elevated levels of psychopathy are associated with a higher willingness to harm others and a lower willingness to maximize other people's happiness and well-being.

#### Interpreting the cultural context of the OUS

This last finding gives us confidence that these results can be extrapolated to other populations because our study's results are highly consistent with Kahane's results. However, caution must be exercised when generalizing these results. Henrich et al. (2010) crucially discuss how a majority of psychology research results are derived from studies on WEIRD (Western, educated, industrialized, rich, and democratic) populations, which rather should be validated also in a variety of cultural contexts for a true generalization and not a culturally specific one. That is, as so-called WEIRD countries are the main producers of scientific research, studies are often marked by cultural, economic, and political similarities, which can lead to significant biases, especially in moral psychology research. For instance, it has been identified that a preponderance of research results in moral psychology have been derived from a few major reference groups (e.g., US students) possessing notable differences with other cultures, which challenges the generalizable veracity of results. This could potentially explain why the validation of the OUS in Turkish (Filiz & Hasan, 2021) uncovered significant differences between the Turkish population and populations living in WEIRD countries such as France and the United States. For example, the results of the Turkish study showed that the OUS-IB was positively correlated with psychopathy and negatively correlated with empathic concern, whereas the OUS-IH was negatively correlated with psychopathy and positively correlated with empathic concern. These findings suggest that substantial inter-individual differences exist, which were not observed in our study due to the use of a WEIRD sample. Therefore, it would be crucial to conduct further validations of the OUS in non-WEIRD countries in order to ensure the generalizability of the results. However, also crucially, WEIRD degree or distance and its association to morality can be more finely assessed as a continuous score (see Muthukrishna et al. 2020) that may further differentiate various countries or their link to morality responses. Given these considerations, it is possible that the differences between our results and the Turkish validation of the OUS can be attributed to differences in the sample populations. As our study involved a diverse range of participants, including those who were not students, it may have provided a more representative sample than the Turkish study, which was limited to a student population.

To conclude, our research findings support that the French OUS has strong psychometric validity. However, this scale seems to be sensitive to cultural differences, as demonstrated by the results of the Turkish translation. This is not surprising because the principles that are prioritized in a moral situation vary depending on the culture (Bentahila et al. 2021). Many studies have shown that culture can shape morality. Snarey (1985) demonstrated that an individual's level of morality is linked to their capacity for reasoning (relative to the Educated criterion of the WEIRD), thus, non-WEIRD societies may have a less developed moral framework compared to their WEIRD counterparts. The study conducted by Awad et al. (2018) and Awad et al. (2020) known as the “moral machine” experiment provides valuable insights into these intercultural differences. The moral machine experiment aimed to explore people's preferences in moral dilemmas involving autonomous vehicles. Participants from various cultural backgrounds were presented with scenarios where an autonomous vehicle had to make a choice that would potentially harm either the passengers inside the vehicle or pedestrians outside. The results of the study revealed distinct patterns based on cultural differences. In Eastern countries such as China and Japan, for instance, there was a stronger preference for protecting the elderly. On the other hand, in Southern countries like those in Latin America, people tended to prioritize individuals of higher social status. In Western countries like Canada and the United States, participants exhibited a preference for inaction rather than action in moral machine dilemmas.

Further elucidating these cultural variances, Atari et al. (2023) conducted a study investigating cross-cultural variations in the endorsement of moral foundations. They sought to build on the principles set by the founders of the moral foundations theory (MFT) (Haidt & Joseph, 2004; Graham et al., 2013). The research was based on the Moral Foundations Questionnaire-2 (MFQ-2) and spanned 25 nations, revealing different moral foundations across these cultures. For instance, Atari and colleagues found that purity and loyalty, two moral foundations, were least endorsed in WEIRD nations like France, but were significantly more salient in non-WEIRD cultures such as Egypt and Morocco. To understand how cultures can be compared to each other, Muthukrishna et al. (2020) developed and validated a tool to measure the psychological and cultural distance between societies. Their work culminated in the creation of a WEIRDness cultural distance scale, offering the ability to gauge how distanced a population is from WEIRD societies, typically using the United States as the point of comparison (e.g., Belgium and France tend to be very close). This tool allows for more nuanced exploration of how the cultural and psychological distance from WEIRD societies can influence a population's moral foundations. Therefore, the cultural parameter should be taken into account when validating scales in other languages, especially in the case of translations intended for WEIRD countries.

### Limitations and future directions

One potential limitation of this study is the lack of test–retest reliability data, as only a single data collection point was used. However, the similarity of our findings to those of Kahane et al. (2018) provides some confidence in the robustness of our analysis. Regarding the study's data, we found relatively weak *R*^2^ values in our regression analyses. This suggests that the model may not fully capture all the factors that contribute to the outcomes being studied, and additional variables may need to be investigated in order to better understand these factors. Another limitation of our study is that for the convergent analysis, we were not able to include the entirety of questionnaires used in the original Kahane et al. study due to the unavailability of validated French versions, and so were limited to those already validated: empathic concern, psychopathy and need for cognition. In this same respect, The Identification with All Humanity Scale (IWAH, McFarland et al., 2012) was not included in this study because it would require validation in French. As a result, some aspects of the OUS were not considered in our study, such as the relationship between utilitarian tendencies and moral attitudes (e.g., prosocial intentions) or the ideology of the participants (e.g., religious belief). To address this limitation, future research could utilize the full range of materials used in the original study. Finally, an additional constraint of our research is the gender imbalance within our sample which may introduce a bias in our results, as gender has been shown to influence various aspects of moral judgment and attitudes. However, we note that we performed additional subsampling analyses to match gender numbers in our groups, and confirmed that they led to similar results in our factor and regression analyses. Despite these nuances, the imbalance in our sample means that we must be cautious when generalizing our findings to all genders. Future research could benefit from ensuring a more balanced sample to further clarify the role of gender in moral judgments.

In future research, it would be important to continue investigating morality through a comparison of different scales in order to better understand the areas of overlap between them. In fact, researchers such as Paruzel-Czachura and Farny (2023) have already begun to explore these questions by studying the link between psychopathy and other tools such as the OUS, the CNI model (Gawronski et al., 2017), and trolley dilemmas (Foot, 1967; Thomson, 1976). Additionally, Paruzel-Czachura and Charzyńska (2022) have observed a link between the Moral Foundations Test and the OUS, further demonstrating the usefulness of comparing different scales to gain a more complete understanding of morality. However, there is still much to be done to disentangle the complex relationships between morality and these different measurement tools. Future studies could also focus on the application of these tools in different cultural contexts in order to determine their applicability and validity in diverse settings. This is particularly pertinent as studies have shown that participants provided different responses to moral dilemmas depending on the language used, as evidenced in studies by Białek et al. (2019) and Costa et al. (2014). Białek et al. (2019) explored this aspect in depth, finding that foreign language reduced sensitivity to consequences and norms without affecting general action tendencies when faced with moral dilemmas. Ultimately, the results suggest that people are less concerned about morality when they deal with moral dilemmas in a foreign language. These findings underscore the importance of ensuring participants engage with surveys in their native language, as some studies demonstrate that moral decision-making, particularly utilitarian perspectives, can be influenced by the language used. While the use of a foreign language impacts moral decision-making, it is also crucial to consider the internal diversity of the speakers of the same language. Thus, considering linguistic homogeneity does not necessarily imply cultural uniformity, variations in moral perception might emerge even among speakers of the same language hailing from diverse cultural backgrounds. For instance, it would be relevant to devise specific attitude scales for distinct Francophone populations. This research aim would be a valuable addition, as it would enable a better understanding of intercultural differences.

## Data Availability

We have included a data availability statement in the revised manuscript to comply with the journal's preferences.
